# Pharmacological Effects of Urolithin A and Its Role in Muscle Health and Performance: Current Knowledge and Prospects

**DOI:** 10.3390/nu15204441

**Published:** 2023-10-19

**Authors:** Haotian Zhao, Ge Song, Hongkang Zhu, He Qian, Xinliang Pan, Xiaoneng Song, Yijie Xie, Chang Liu

**Affiliations:** 1 Department of Physical Education, Jiangnan University, Wuxi 214122, China; haotianzhao@jiangnan.edu.cn; 2 School of Food Science and Technology, Jiangnan University, Wuxi 214122, China; 7210112118@stu.jiangnan.edu.cn (H.Z.); amtf168168@126.com (H.Q.); 3 School of Sport Science, Beijing Sport University, Beijing 100084, China; songge638@bsu.edu.cn (G.S.); panxinliang@bsu.edu.cn (X.P.); 4 Affiliated Hospital of Jiangnan University, Wuxi 214062, China

**Keywords:** Urolithin A, muscle health, muscle performance, pharmacology, review

## Abstract

Urolithin A (UA) is a naturally occurring compound derived from the metabolism of gut microbiota, which has attracted considerable research attention due to its pharmacological effects and potential implications in muscle health and performance. Recent studies have demonstrated that Urolithin A exhibits diverse biological activities, encompassing anti-inflammatory, antioxidant, anti-tumor, and anti-aging properties. In terms of muscle health, accumulating evidence suggests that Urolithin A may promote muscle protein synthesis and muscle growth through various pathways, offering promise in mitigating muscle atrophy. Moreover, Urolithin A exhibits the potential to enhance muscle health and performance by improving mitochondrial function and regulating autophagy. Nonetheless, further comprehensive investigations are still warranted to elucidate the underlying mechanisms of Urolithin A and to assess its feasibility and safety in human subjects, thereby advancing its potential applications in the realms of muscle health and performance.

## 1. Introduction

In recent years, there has been a substantial increase in the recognition of the critical importance of both health and athletic performance [1,2]. The pursuit of muscle health and optimal athletic performance is no longer confined solely to athletes or fitness enthusiasts but has become a pervasive goal among the general population, who strive for a healthier and more active lifestyle [3,4]. Consequently, there is a widespread demand for strategies to enhance muscle health and improve athletic performance, rendering this field an area of profound discussion and extensive research.

**Figure 1:**
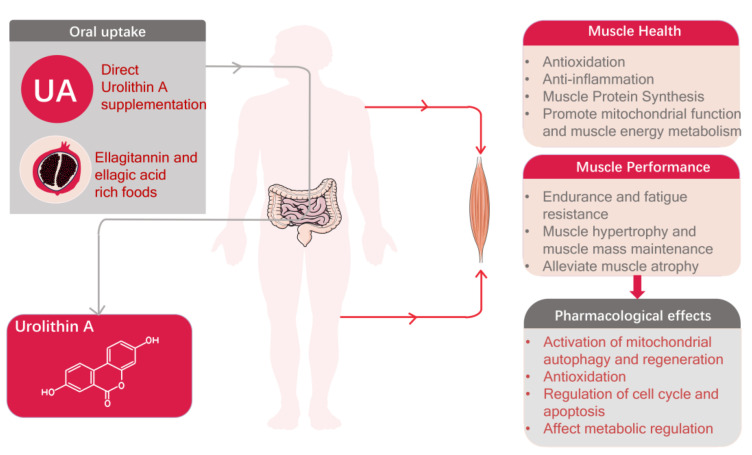
Pharmacological effects of Urolithin A and its role in promoting muscle health and enhancing athletic performance.

Urolithin A, a naturally occurring compound derived from dietary sources, has swiftly emerged as a prominent subject of investigation in the context of muscle health and performance [5,6,7]. Existing evidence supports the potential of Urolithin A in facilitating muscle cell proliferation and augmenting muscle function (Figure 1) [8,9,10]. However, the understanding of the mechanisms of action and potential applications of Urolithin A as it relates to muscle health and athletic performance is still in its infancy.

The present review aims to provide a comprehensive survey and analysis of current research on the impact of Urolithin A on muscle health and performance, as well as suggest directions for future research. The topic will be examined from multiple perspectives, consolidating and assessing existing experimental outcomes to unravel the correlation between Urolithin A, muscle health, and corresponding biological processes and molecular mechanisms. Furthermore, potential areas of application of Urolithin A, including its feasibility as a dietary supplement or pharmaceutical product, will be discussed. This paper, grounded in an extensive literature review and systematic analysis, seeks to synthesize current research findings to offer fresh insights into the role of Urolithin A in muscle health and performance. It is anticipated that these insights may provide valuable guidance for further exploration into the functional mechanisms of Urolithin A, ultimately facilitating the development of innovative strategies to enhance muscle health.

## 2. Urolithin A’s Sources in the Diet

Urolithin A, a natural metabolite derived from ellagitannins, is biosynthesized by the gut microbiota [11,12], It is a type of compound known as urolithins, which are present in pomegranates and certain other fruits and nuts such as strawberries, walnut kernels, and peanuts. Ellagitannins serve as precursors to urolithins and undergo microbial metabolism within the gastrointestinal tract to produce Urolithin A [7]. Pomegranate, walnut, and almond stand out as the primary and richest dietary sources of Urolithin A [13,14]. Pomegranate peel and seeds contain abundant polyphenolic compounds, including Urolithin A formed through metabolism by the gut microbiota. Walnut is also considered a dietary source of Urolithin A, as it is rich in polyphenolic compounds and anthocyanins that may interact with gut microbiota during digestion and potentially generate Urolithin A. Almond is another potential dietary source of Urolithin A. It contains polyphenolic compounds, particularly flavonoids and anthocyanins, which can be converted into Urolithin A upon interaction with gut microbiota. Blueberry, recognized for its antioxidant-rich content, including various polyphenolic compounds, which may also include Urolithin A [15]. However, research on the content of Urolithin A in blueberry remains limited, necessitating further studies to confirm its source and concentration [16].

## 3. Metabolism and Bioavailability of Urolithin A in the Body

### 3.1. Gut Microbiota Metabolism

**Figure 2:**
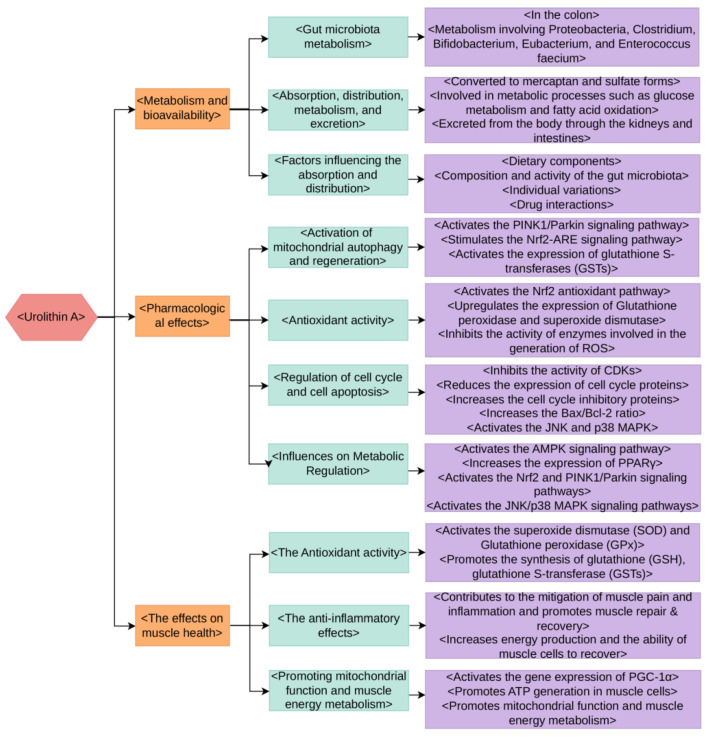
The metabolism, bioavailability, pharmacological effects, and impact on muscle health of Urolithin A.

The production of Urolithin A is largely dependent on the metabolic activities of gut microbiota [7]. During the process of digestion, several polyphenolic compounds found in food such as tannins and anthocyanins, undergo enzymatic reactions and metabolic conversions facilitated by microbial enzymes, culminating in the production of Urolithin A [17]. This metabolism predominantly takes place in the colon, which houses a variety of microbial groups. These groups, including Proteobacteria, Clostridium, Bifidobacterium, Eubacterium, and Enterococcus faecium possess distinct metabolic functions that play a part in the production of Urolithin A (Figure 2) [18,19,20,21,22].

### 3.2. Absorption, Distribution, Metabolism, and Excretion

Following absorption in the intestines, Urolithin A enters the circulatory system and is transported to various tissues and organs through the bloodstream [11,23]. Studies have indicated that in the human body, Urolithin A primarily exists in a free form rather than being bound to other molecules [24]. Once inside the cell, Urolithin A undergoes further metabolism through multiple metabolic pathways [25]. One prominent pathway involves the conversion of Urolithin A into its mercaptan and sulfate forms, facilitated by metabolic enzymes present in the liver [26]. Furthermore, some studies have suggested that Urolithin A may participate in metabolic processes such as glucose metabolism and fatty acid oxidation. Urolithin A and its metabolites can be excreted from the body through the kidneys and intestines [25]. A portion of Urolithin A is further metabolized into other compounds within the intestines before being eliminated through feces [24]. These processes may be influenced by factors such as intestinal permeability, renal function, and individual characteristics.

It is important to note that the metabolism and utilization of Urolithin A may vary among individuals, depending on their intestinal microbial composition, metabolic capacity, genetic factors, dietary habits, and other physiological states [8,27]. Furthermore, the bioavailability of Urolithin A may also be influenced by the dietary source, intake level, and tissue specificity [28]. Although some insights into the metabolic mechanisms and bioavailability of Urolithin A have been gained, further research is still needed to comprehensively understand its behavior and effects in the human body, as well as its associations with relevant health effects.

### 3.3. Factors Influencing the Absorption and Distribution of Urolithin A

#### 3.3.1. Dietary Components

The absorption and distribution of Urolithin A can be influenced by compounds present in food. For instance, the consumption of polyphenolic-rich foods, such as pomegranate, walnuts, almonds, and others, can supply an abundance of precursor molecules for intestinal microbial metabolism, facilitating the generation of Urolithin A [29,30,31]. Moreover, certain components in food may compete with Urolithin A for absorption within the gastrointestinal tract, consequently impacting its bioavailability [24].

#### 3.3.2. Composition and Activity of the Gut Microbiota

The composition and activity of an individual’s intestinal microbiota play a pivotal role in Urolithin A metabolism and absorption [32]. The gut microbial communities in different individuals may exhibit distinct capabilities in converting polyphenolic compounds present in food into Urolithin A [33]. Therefore, interindividual variations could potentially impact the bioavailability of Urolithin A.

#### 3.3.3. Individual Variations

Individual variations can also have an impact on the absorption and distribution of Urolithin A. Genetic factors, for instance, can lead to interindividual differences in the activity and expression of metabolic enzymes, which can affect the rate of Urolithin A metabolism. Additionally, variations in intestinal permeability, the health status of the digestive system, and other physiological states may impact the absorption and distribution of Urolithin A [34].

#### 3.3.4. Drug Interactions

Currently, there is a lack of direct research on the effects of drugs on the metabolism and clearance processes of Urolithin A. However, certain drugs may interact with Urolithin A and affect its absorption and metabolism. For example, specific drugs may competitively inhibit or induce metabolic enzymes, interfering with the metabolism and clearance of Urolithin A. Drug interactions with cytochrome P450 (CYP450) enzymes in the liver, for instance, can competitively inhibit or induce these enzyme, thereby disrupting the metabolism and clearance of Urolithin A [35]. Commonly used antibiotics, antifungal drugs, and anticancer drugs have been reported to interact with CYP450 enzymes [36]. Hepatic enzyme inducers, such as rifampicin and carbamazepine, can increase the activity of certain metabolic enzymes in the liver, potentially accelerating the metabolism of Urolithin A and reducing its half-life in the body [37]. Certain drugs, such as clarithromycin, can inhibit the activity of metabolic enzymes in the liver, slowing down the metabolism and clearance processes of Urolithin A and leading to its accumulation in the body [38].

Although the factors mentioned above may influence the absorption and distribution of Urolithin A, further research is essential to gain a more precise understanding of their specific effects on the metabolism and bioavailability of Urolithin A. Continued studies will contribute to a more comprehensive comprehension of Urolithin A’s behavior and effects, offering more accurate guidance for its application in health management.

## 4. Pharmacological Effects of Urolithin A

### 4.1. Activation of Mitochondrial Autophagy and Regeneration

Mitochondrial autophagy, also known as mitophagy, refers to the selective degradation of mitochondria through the autophagic process. This process typically occurs in mitochondria that are damaged or defective due to stress. Mitochondrial autophagy plays a pivotal role in maintaining cellular health by facilitating the turnover of mitochondria and preventing the accumulation of dysfunctional ones, which could otherwise lead to cellular degeneration. The regulation of mitochondrial autophagy is mediated by proteins such as PINK1 and Parkin [39,40,41].

Apart from its function in selectively eliminating damaged mitochondria, mitochondrial autophagy is essential for adjusting the mitochondrial population to meet changing cellular metabolic demands, ensuring mitochondrial turnover and homeostasis. Research has demonstrated that Urolithin A activates the PINK1/Parkin signaling pathway, which is involved in mitochondrial quality control. Consequently, this activation promotes the selective aggregation, degradation, and removal of damaged mitochondria [42].

Furthermore, research has illuminated Urolithin A’s capacity to enhance mitochondrial autophagy by activating the expression of glutathione S-transferases (GSTs). GSTs are pivotal detoxifying enzymes intricately related to antioxidant capacity. Urolithin A stimulates the Nrf2-ARE signaling pathway, subsequently upregulating the expression of GSTs, thereby enhancing cellular autophagy and mitochondrial quality control [43].

In summary, Urolithin A participates in regulating mitochondrial autophagy and quality control by activating the PINK1/Parkin and glutathione S-transferases signaling pathway.

### 4.2. Antioxidant Activity

Antioxidant activity refers to the capacity to inhibit oxidation, a chemical reaction that typically involves the generation of free radicals, often through autoxidation processes. Urolithin A exhibits antioxidant activity through multiple mechanisms. Firstly, it possesses the ability to directly scavenge free radicals by capturing and neutralizing reactive oxygen species such as peroxy radicals and superoxide radicals, thus reducing cellular oxidative stress [44]. Secondly, Urolithin A can activate the Nrf2 antioxidant pathway [45] and upregulate the expression of various antioxidant enzymes such as Glutathione peroxidase and superoxide dismutase, thereby enhancing cellular antioxidant defense capacity. Additionally, Urolithin A has been reported to inhibit enzyme activities involved in ROS generation, thereby reducing the occurrence of oxidative stress and protecting cells from oxidative damage [46].

### 4.3. Regulation of Cell Cycle and Cell Apoptosis

Research has shown that Urolithin A can regulate the cell cycle and induce apoptosis [47]. In terms of cell cycle regulation, Urolithin A can impact cell cycle-related protein kinase complexes, such as cyclin-dependent kinases (CDKs) and cyclins. It inhibits the activity of CDKs and reduces the expression of cell cycle proteins such as cyclin D1, leading to cell cycle arrest in the G1 phase [47]. Additionally, Urolithin A can regulate the cell cycle by increasing the levels of cell cycle inhibitory proteins, such as p21 and p27 [48,49].

Regarding apoptosis, Urolithin A can induce cell death through multiple pathways. Studies have found that Urolithin A increases the Bax/Bcl-2 ratio, resulting in the loss of mitochondrial membrane potential, release of cytochrome c, and activation of caspase cascades, ultimately leading to cell apoptosis [49]. Furthermore, Urolithin A can activate the JNK (c-Jun *N*-terminal kinase) and p38 MAPK (mitogen-activated protein kinase) signaling pathways, further promoting apoptosis [50].

### 4.4. Influences on Metabolic Regulation

Urolithin A participates in the regulation of multiple metabolic pathways. Research has shown that Urolithin A can activate the adenosine monophosphate-activated protein kinase (AMPK) signaling pathway, which is a pivotal regulator of energy metabolism [51]. Activation of AMPK promotes fatty acid oxidation and insulin sensitivity while reducing fatty acid synthesis and gluconeogenesis [51,52]. Additionally, Urolithin A can modulate the activity of peroxisome proliferator-activated receptor gamma (PPARγ) [53], a crucial transcription factor involved in adipocyte differentiation, glucose metabolism, and cholesterol metabolism, among other processes [54]. Studies have indicated that Urolithin A can increase the expression of PPARγ and transcription of its downstream target genes, thereby promoting fatty acid oxidation, improving insulin sensitivity, and reducing cholesterol synthesis and absorption [53].

In conclusion, Urolithin A regulates the cell cycle by activating the Nrf2 and PINK1/Parkin signaling pathways, inhibiting ROS production, and modulating the activity of CDKs and cyclins. It also induces cell apoptosis through the modulation of the Bax/Bcl-2 ratio and activation of the JNK/p38 MAPK signaling pathways. Additionally, Urolithin A’s regulatory effects involve the activation of metabolic regulators such as AMPK and PPARγ. These molecular mechanisms collectively contribute to the various cellular and biological effects of Urolithin A. Further research is needed to unravel the detailed mechanisms of Urolithin A and its interactions with other signaling pathways.

## 5. The Effects of Urolithin A on Muscle Health

### 5.1. The Antioxidant Activity of Urolithin A

Oxidative stress results from an excess production of free radicals and reactive oxygen species (ROS) [55]. During exercise, especially strenuous activities, the body is more susceptible to generating an excessive amount of ROS, and these reactive substances can inflict damage on cellular structures and functions. Urolithin A, functioning as an antioxidant, can neutralize free radicals and mitigate their harmful effects to cells. This action can lower the level of oxidative stress within muscle cells and protect cellular structures and functions, thereby promoting muscle health.

Urolithin A may also be able to activate critical antioxidant enzymes in the body, such as superoxide dismutase (SOD) and Glutathione peroxidase (GPx). These enzymes actively scavenge free radicals and peroxides within cells, reducing the extent of oxidative stress and contributing to muscle health. Additionally, Urolithin A can further promote the synthesis of certain antioxidant molecules, such as glutathione (GSH), glutathione S-transferase, etc. [44]. These molecules play a vital role in regulating oxidative stress and preserving the health of muscle cells.

Furthermore, the antioxidant properties of Urolithin A can reduce the occurrence of inflammatory reactions [56]. Oxidative stress and inflammation are often intertwined, mutually exacerbating each other. Urolithin A may attenuate the intensity of inflammatory responses by alleviating oxidative stress, thereby exerting a positive impact on muscle health.

### 5.2. The Anti-Inflammatory Effects of Urolithin A

Urolithin A possesses significant anti-inflammatory effects, which are instrumental for muscle recovery. During exercise and physical exertion, muscles undergo a certain degree of damage, triggering an inflammatory response [57]. Moderate inflammation constitutes a natural process of the body’s repair, but excessive or prolonged inflammation may impair muscle recovery and growth. Research has indicated that Urolithin A can inhibit the inflammatory response and alleviate inflammation-induced damage [58]. It exerts these anti-inflammatory effects through multiple mechanisms, including inhibiting the release of inflammatory mediators, suppressing the activation of inflammatory signaling pathways, and reducing oxidative stress. By suppressing the inflammatory response, Urolithin A contributes to the mitigation of muscle pain and inflammation and promotes muscle repair and recovery [59]. This is particularly important for post-exercise muscle recovery as it expedites the healing process of muscle cells, reducing muscle soreness and discomfort, and improving muscle function and performance [57].

In addition, Urolithin A is also able to promote mitochondrial function and muscle energy metabolism, which has a positive impact on muscle recovery and performance. By enhancing mitochondrial function, Urolithin A can increase energy production and muscle cells’ ability to recover, thereby accelerating the muscle recovery process. Overall, the anti-inflammatory effects of Urolithin A are crucial for muscle recovery. It not only alleviates inflammation caused by exercise but also promotes the repair and energy metabolism of muscle cells, ultimately enhancing muscle recovery ability and performance level.

### 5.3. The Role of Urolithin A in Promoting Mitochondrial Function and Muscle Energy Metabolism

Urolithin A plays a substantial role in promoting mitochondrial function and muscle energy metabolism [51]. Mitochondria are the principal organelles responsible for cellular energy production and are crucial for muscle cell function and performance. Research has shown that Urolithin A can enhance mitochondrial function and activity [10]. It achieves this by activating gene expression similar to PGC-1α (transcriptional coactivator peroxisome proliferator-activated receptor γ coactivator-1α), regulating the quantity and quality of mitochondria. PGC-1α is a pivotal regulatory transcription factor involved in mitochondrial biogenesis, the formation of respiratory chain complexes, and processes such as mitochondrial fission and fusion [60,61].

Urolithin A also exhibits antioxidant properties, reducing the generation of free radicals and protecting mitochondria from oxidative stress-induced damage. Excessive free radicals production and oxidative stress can damage the structure and function of mitochondria, affecting muscle energy metabolism and recovery capacity. Urolithin A clears free radicals, protects mitochondria from damage, and improves the efficiency of energy production. Furthermore, Urolithin A can promote ATP generation in muscle cells. ATP is the primary energy molecule for muscle cells and is crucial for muscle contraction and exercise performance [62]. Research has found that Urolithin A can promote the formation and enhanced function of mitochondrial respiratory chain complexes, thereby improving the synthesis rate of ATP in muscle cells [10]. By promoting mitochondrial function and muscle energy metabolism, Urolithin A has a positive impact on muscle performance and recovery. It can increase energy supply to muscle cells and improve muscle endurance and strength performance. Additionally, Urolithin A alleviates fatigue caused by exercise and promotes rapid muscle recovery.

In summary, Urolithin A assumes a vital role in muscle performance and recovery by promoting mitochondrial function and muscle energy metabolism. It enhances mitochondrial activity, reduces oxidative stress and free radical generation, and promotes ATP synthesis, thereby improving the energy supply and physical performance in muscle cells.

## 6. The Effects of Urolithin A on Muscle Performance

### 6.1. The Potential Effects of Urolithin A on Endurance and Anti-Fatigue Capacity

Limited research currently supports the potential impact of Urolithin A on endurance and anti-fatigue capacity [9,10]. Although further research is needed, existing evidence suggests that Urolithin A may have a positive role in improving endurance and delaying muscle fatigue, both of which are intricately linked to mitochondrial function [63]. Firstly, Urolithin A can increase the energy supply of muscle cells by promoting mitochondrial function and muscle energy metabolism [7]. Mitochondria are crucial components for cellular energy production, and Urolithin A can enhance mitochondrial activity and ATP synthesis rates [8]. This bears significance for sustained exercise and endurance performance, as it can delay the onset of muscle fatigue and provide prolonged energy support. Secondly, Urolithin A possesses anti-inflammatory properties which can alleviate tissue damage and muscle soreness caused by inflammatory reactions [58]. Excessive or prolonged inflammation can lead to muscle fatigue and reduced endurance. By inhibiting inflammatory responses, Urolithin A helps alleviate muscle pain and discomfort, protects muscles from inflammation-induced injuries, and consequently enhances endurance and anti-fatigue capacity. Additionally, Urolithin A can improve muscle contraction speed and exercise efficiency, which also has a positive impact on endurance and anti-fatigue capacity [8]. This enhancement is achieved by promoting mitochondrial function and regulating muscle cell energy metabolism, enhancing the efficiency and force output of muscle contractions. This means that Urolithin A has the potential to delay the onset of fatigue and improve endurance during prolonged or high-intensity exercise.

It is essential to note that there is currently limited research available regarding the effects of Urolithin A on endurance and anti-fatigue capacity, and most studies have been conducted in vitro or on animals. Therefore, more human studies are needed to validate these potential effects and determine the optimal dosage and duration. However, the current evidence suggests that Urolithin A may hold promise for enhancing endurance and anti-fatigue capacity, especially with continuous supplementation.

### 6.2. The Effects of Urolithin A on Muscle Hypertrophy and Maintenance of Muscle Mass

Urolithin A may have a positive impact on muscle hypertrophy and maintenance of muscle mass. It exerts its effects through multiple pathways, including promotion of muscle cell repair and growth, enhancement of mitochondrial function and energy metabolism, and regulation of protein synthesis and degradation processes [8,53]. Firstly, Urolithin A can promote muscle cell repair and growth. During exercise and training, muscles undergo damage that requires repair and growth for muscle hypertrophy to occur [64]. Urolithin A provides a better environment for muscle cell repair and growth by promoting mitochondrial function and increasing ATP synthesis [65]. This can increase the cross-sectional area of muscle fibers, muscle protein content, and muscle strength, thus promoting muscle hypertrophy. Secondly, the regulation of protein synthesis and degradation processes by Urolithin A is also crucial for muscle hypertrophy and maintenance of muscle mass. It can increase the rate of protein synthesis within muscle cells and inhibit protein degradation processes [53]. This allows muscle cells to more effectively synthesize new proteins and maintain muscle mass. Additionally, Urolithin A can improve muscle endurance and exercise performance by promoting mitochondrial function and muscle energy metabolism [66]. This may lead to longer and more intense training sessions, further promoting muscle hypertrophy and the maintenance of muscle mass.

It should be noted that current research is still limited and mostly conducted in vitro or animal experiments. More human studies are needed to validate the potential effects of Urolithin A on muscle hypertrophy and the maintenance of muscle mass and to determine the optimal dosage and duration. However, based on current evidence, Urolithin A may have a positive impact on promoting muscle hypertrophy and maintaining muscle mass, particularly when used in combination with appropriate exercise and dietary plans [67].

## 7. The Signaling Pathways and Mechanisms of Action of Urolithin A in Muscle

### 7.1. The Interaction of Urolithin A with Key Signaling Pathways Involved in Muscle Health and Performance

#### 7.1.1. AMPK Pathway

**Figure 3:**
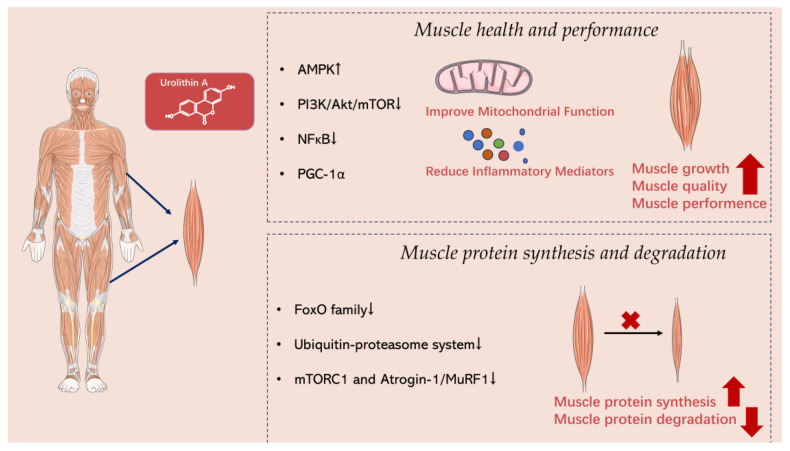
The signaling pathways and mechanisms of action of Urolithin A in muscle.

Urolithin A has been found to activate the AMPK (adenosine monophosphate-activated protein kinase) pathway [51]. By activating AMPK, Urolithin A can increase mitochondrial biogenesis and enhance fatty acid oxidation and glycogen synthesis, ultimately improving muscle energy supply and metabolism and enhancing muscle performance (Figure 3) [68]. The literature demonstrates that Urolithin A activates AMPK through a cascade involving the activation of SIRT3 and, subsequently, LKB1 [69].

#### 7.1.2. mTOR Pathway

The mTOR (mammalian target of rapamycin) pathway plays a key role in regulating muscle protein synthesis and cell growth [70,71,72,73]. Research has found that Urolithin A can regulate protein synthesis and growth of muscle cells by inhibiting specific regulatory factors within the mTOR signaling pathway, such as PI3K (phosphoinositide 3-kinase) and Akt (protein kinase B) [74,75]. This regulation supports muscle growth and maintains muscle quality [70]. The literature has demonstrated that Urolithin A inhibits mTOR by first inhibiting PI3K and then AKT.

#### 7.1.3. NF-κB Pathway

The NF-κB (nuclear factor kappa-light-chain-enhancer of activated B cells) pathway is implicated in the regulation of inflammatory responses [76,77]. Urolithin A has been found to inhibit the activation of NF-κB and reduce the production of inflammatory mediators [78,79]. By inhibiting the NF-κB pathway, Urolithin A can mitigate muscle damage and pain caused by inflammatory responses, thereby promoting muscle recovery and health [44]. The literature indicates that Urolithin A blocks the NF-κB/STAT1 Axis through the inactivation of TLR3/TRIF signaling.

#### 7.1.4. PGC-1α Pathway

PGC-1α is an important transcription coactivator that regulates mitochondrial biogenesis and cellular energy metabolism [60,61]. Research has found that Urolithin A can enhance the expression and activation of PGC-1α, leading to improved mitochondrial function and increased mitochondrial quantity [80,81,82]. This enhancement is crucial for ensuring an adequate muscle energy supply and supporting endurance performance [83]. The scientific literature provides evidence that Urolithin A activates PGC-1α through a cascade that includes the activation of SIRT3, followed by the activation of LKB1 and AMPK.

### 7.2. The Regulatory Effects of Urolithin A on Muscle Protein Synthesis and Degradation

#### 7.2.1. FoxO Family

The FoxO (forkhead box O) family is a group of transcription factors that are involved in regulating the synthesis and degradation of muscle proteins [84,85]. Studies have found that Urolithin A can inhibit the degradation of muscle proteins by suppressing the activation of FoxO [86]. This is primarily achieved by inhibiting the nuclear activity of FoxO and reducing their regulation of specific protein degradation pathways.

#### 7.2.2. Ubiquitin-Proteasome System

The ubiquitin-proteasome system stands as one of the main pathways responsible for the degradation of intracellular proteins [87,88,89]. Studies have found that Urolithin A can attenuate the degradation of muscle proteins by inhibiting the activity of the ubiquitin–proteasome system [90]. This inhibition may be achieved by influencing the activity of specific ubiquitin ligases or proteasomes.

#### 7.2.3. mTORC1 and Atrogin-1/MuRF1

mTORC1 (mammalian target of rapamycin complex 1) and Atrogin-1/MuRF1 (muscle-specific RING finger protein 1 and 2) are pivotal molecules involved in muscle protein synthesis and degradation [91,92,93,94]. Studies have shown that Urolithin A can regulate muscle protein synthesis and degradation by inhibiting the activity of mTORC1 [75,95] and the expression of Atrogin-1/MuRF1 [23].

## 8. Clinical Research Limitations and Future Perspectives

Overall, there is limited research available on the effects of Urolithin A in both physiological conditions and muscular pathologies. Key findings from existing studies are summarized below:

Effects of Urolithin A in Physiological Conditions:

1. Enhanced Muscle Function: Urolithin A has demonstrated the ability to improve muscle function in individuals with normal muscle health. It achieves this by promoting mitochondrial biogenesis, which enhances the energy production capacity of muscle cells. This enhancement results in improved muscle endurance, strength, and overall performance during physical activities [39,40,42].

2. Delaying Muscle Fatigue: Urolithin A shows promise in delaying the onset of muscle fatigue during prolonged or high-intensity exercise. By enhancing mitochondrial function and energy metabolism in muscle cells, it provides sustained energy support, enabling individuals to engage in longer and more intense training sessions [9,10,39,40,41,42].

3. Muscle Protein Synthesis: Urolithin A can stimulate muscle protein synthesis, contributing to muscle growth and maintenance. This effect is particularly beneficial for individuals looking to build and preserve muscle mass, such as athletes and fitness enthusiasts [8,53].

4. Antioxidant Defense: Urolithin A exhibits potent antioxidant properties, safeguarding muscle cells from oxidative stress and damage induced by free radicals and reactive oxygen species. This antioxidant defense is crucial for maintaining muscle health and function under normal conditions [44,45,46].

Effects of Urolithin A in Pathologies of the Muscular System:

1. Muscle Atrophy: In pathological conditions characterized by muscle atrophy, such as certain neuromuscular diseases or extended periods of immobility, Urolithin A may offer potential benefits [42,47]. It can stimulate muscle protein synthesis while inhibiting protein degradation pathways, helping to preserve muscle mass and mitigate muscle wasting [48,49].

2. Mitochondrial Dysfunction: Some muscular pathologies involve mitochondrial dysfunction, leading to reduced energy production and muscle weakness [51]. Urolithin A’s capacity to enhance mitochondrial function and biogenesis holds promise in alleviating these issues, thereby improving muscle energy supply and overall function [39,40,42].

3. Inflammatory Conditions: Muscular pathologies often involve inflammation, which can exacerbate muscle damage and hinder recovery [57]. Urolithin A’s anti-inflammatory properties can be valuable in reducing the intensity of inflammatory responses, promoting muscle recovery, and enhancing overall muscle health in conditions characterized by chronic inflammation [58,59].

4. Oxidative Stress: Muscular pathologies can result in increased oxidative stress within muscle cells. Urolithin A’s antioxidant activity helps mitigate oxidative damage, protecting muscle tissue and supporting its recovery and regeneration [10,44,45,46,62].

While the precise mechanisms and efficacy of Urolithin A in specific muscular pathologies require further research, its potential benefits in both physiological conditions and certain pathological states make it a compelling subject for future investigations and potential clinical applications.

Currently, most clinical studies related to Urolithin A are still in their early stages, characterized by relatively small sample sizes. Due to these limitations in study size, further research is required to confirm the reliability and widespread applicability of the results. Additionally, most studies have relatively short periods, and the long-term effects and safety of Urolithin A usage have not been adequately evaluated. Conducting long-term follow-up studies will provide insights into the long-term effects and potential risks of using Urolithin A. Furthermore, it is not yet clear what the optimal dosage and timing of Urolithin A should be. Different studies have used varying doses and regimens, making it challenging to compare and infer the most effective usage. Further research is needed to determine the optimal dosage and ideal administration strategy.

Additionally, the bioavailability and metabolism mechanisms of Urolithin A require further in-depth research. Investigating its absorption, distribution, and metabolic pathways within the body will facilitate better guidance on its use and effects. Moreover, there may be individual differences and heterogeneity in the human response to Urolithin A. Therefore, research needs to consider factors such as diet, genetics, and the environment that may interfere with and influence the effects of Urolithin A, to more accurately assess its mechanisms of action and effects. There are already some commercially available products related to Urolithin A on the market. However, the quality and purity of these products may vary, so it is important to ensure the acquisition of high-quality products from reliable sources and exercise appropriate dosage control.

In summary, although Urolithin A exhibits promising potential and positive effects, current research still faces challenges and limitations. Future studies need to address these issues and conduct larger-scale, long-term follow-up clinical studies to further validate the mechanisms of action, dosage effects, and safety of Urolithin A.

## 9. Future Research Directions and Potential Applications of Urolithin A in Exercise Science

Further long-term follow-up studies are imperative to gain a comprehensive understanding of the effects of prolonged use of Urolithin A on muscle health, exercise performance, and recovery. These studies can assess the long-term safety, efficacy, and enduring impact of Urolithin A on muscle function and recovery. Additionally, expanding the scale of research and conducting larger clinical trials will aid in confirming the effects of Urolithin A. This will aid in determining the optimal dosage, timing, and duration of Urolithin A usage, as well as evaluating its applicability to different populations and types of exercise. Further research is still required to investigate the mechanisms of action of Urolithin A in critical biological processes such as muscle metabolism, mitochondrial function, and protein synthesis/degradation. Through these studies, a better understanding of the interaction between Urolithin A and crucial signaling pathways, as well as how it regulates muscle biological processes, can provide more mechanistic explanations for its application in exercise.

Research exploring the effects of Urolithin A in different populations such as the elderly, athletes, and individuals with muscle injuries or diseases can determine the applicability of Urolithin A in specific populations and understand its potential benefits and safety in these populations. Studies conducting exploratory studies on the interactions of Urolithin A with other nutrients (such as proteins, amino acids, etc.) or medications can evaluate the synergistic effects of Urolithin A with other interventions and determine the optimal combination strategies to enhance muscle health and exercise performance. Additionally, exploring the impact of Urolithin A on muscle gene regulation through gene expression analysis and genetic studies can elucidate the molecular mechanisms of Urolithin A in muscle health and exercise adaptation, providing valuable information for personalized exercise interventions.

In conclusion, future research should continue to delve deeper into the potential applications of Urolithin A in sports and exercise science. These endeavors will further validate its mechanisms of action, dosage effects, and safety considerations while taking into account the applicability to different populations and individual variances. Such studies will contribute to providing more specific and reliable guidance for the use of Urolithin A in sports.

## 10. Conclusions

In conclusion, key findings regarding Urolithin A in muscle health and performance encompass its regulatory effects on promoting muscle protein synthesis while inhibiting degradation, its interactions with crucial signaling pathways, potential enhancement of endurance and fatigue resistance, as well as its anti-inflammatory properties. These findings establish a solid scientific foundation for the potential application of Urolithin A in improving muscle health, promoting muscle growth, and enhancing exercise performance. However, further research is needed to validate these findings and to explore the safety, optimal usage, and suitable populations for Urolithin A.
